# Bile acid metabolites and carotid intima media thickness in the Chronic Kidney Disease in Children (CKiD) study

**DOI:** 10.1007/s00467-026-07343-1

**Published:** 2026-05-08

**Authors:** Arthur M. Lee, Mark M. Mitsnefes, Jennifer Roem, Bradley A. Warady, Susan L. Furth, Michelle R. Denburg

**Affiliations:** 1 University of Pennsylvania Perelman School of Medicine, Philadelphia, PA USA; 2 Children’s Hospital of Philadelphia, Philadelphia, PA USA; 3 Cincinnati Children’s Hospital Medical Center, Cincinnati, OH USA; 4 Johns Hopkins Bloomberg School of Public Health, Baltimore, MD USA; 5 Children’s Mercy Kansas City, Kansas City, MO USA; 6 University of Missouri – Kansas City, Kansas City, MO USA

**Keywords:** Chronic kidney disease, Cardiovascular disease, Atherosclerosis, Bile acids

## Introduction

Cardiovascular disease is the leading cause of mortality in children with chronic kidney disease (CKD). Children with CKD can have subclinical atherosclerosis as an early manifestation of cardiovascular disease [1].

Circulating bile acids are a hypothesized mediator of atherosclerosis in CKD [2]. As kidney function declines, circulating bile acid levels become elevated. The composition is shifted towards a greater proportion of secondary bile acids. Bile acids exert toxicity by binding to various receptors expressed in vascular smooth muscle and epithelial cells to affect contractility, dilation, and promote vascular calcification [3]. In adults with CKD, bile acids have associated with increased coronary artery calcification, CKD progression, and mortality [2, 4].

The effect of circulating bile acids on atherosclerotic burden in children with CKD is less studied. Participants in the Chronic Kidney Disease in Children (CKiD) study had increased carotid intima media thickness (CIMT) compared to matched healthy controls [1]. In this study, we sought to confirm the association of bile acids with atherosclerotic disease burden in children with CKD.

## Methods

CKiD is a North American multicenter prospective cohort that enrolled children aged 1 to 16 years with estimated glomerular filtration rate (eGFR) 30–90 ml/min/1.73 m^2^. CKiD study protocols have been previously published [5]. We analyzed data from five sites that participated in the CIMT substudy (2005–2009). Covariables relevant to this analysis include eGFR, hypertension, dyslipidemia, and urine protein–creatinine ratio (UPCR) which have been defined in CKiD publications [6–8].

Protocolized carotid ultrasonography was performed at the 1- (henceforth referred to as baseline), 3-, and 5-year study visits. CIMT measurements were standardized at the Cincinnati Cardiovascular Core Imaging Research Laboratory [1].

Plasma samples from the baseline, 2-year, and 4-year study visits were sent to Metabolon Inc. (Durham, NC) for untargeted metabolomics quantification. Data QC was consistent with previously published NIDDK CKD Biomarkers Consortium consensus [9]. Baseline and follow-up sample profiling were completed in two batches. Analysis plan was designed to avoid direct comparison of baseline and follow-up metabolomics data.

The association between log-transformed bile acid level and log-transformed CIMT were assessed with multivariable models adjusting for age, sex, glomerular CKD etiology, CKD duration, hypertension, dyslipidemia, log-UPCR, serum calcium, serum phosphorus, and log-intact parathyroid hormone (iPTH). For the baseline data, we performed linear regression analyses. For the follow-up data, we performed linear mixed effects modeling.

Bile acids are associated with kidney function, and statistical adjustment for eGFR may mask true biologic effects. We performed the analyses both with and without adjusting for eGFR. Bile acid metabolites violate statistical assumptions for multiple comparisons adjustments: they are highly multicollinear and individual metabolites do not represent unique biologic pathways. We report on significance threshold of both *p* < 0.05 and Benjamini–Hochberg False Discovery Rate < 0.05.

## Results

The study population included 117 CKiD participants with both baseline CIMT measurements and metabolomic profiling. 74% of participants were male, 38% had glomerular CKD etiology, 70% had hypertension, and 51% had dyslipidemia. Participant characteristics reported as median (interquartile range) were age = 13 (9,15) years, CKD duration = 9 (4, 13) years, UPCR = 0.4 (0.1, 0.9), serum calcium = 9.4 (9.2, 9.8) mg/dL, serum phosphorus = 4.5 (3.9, 4.9) mg/dL, iPTH = 40 (26, 57) pg/mL, and eGFR = 53 (37, 70) ml/min/1.73 m^2^.

The analysis examined 27 bile acid metabolites. Table 1 shows bile acid metabolites associated with CIMT in the baseline analyses.

**Table 1:** Adjusted associations of bile acid metabolites with CIMT at baseline—Spearman correlation assessed the relation between bile acids and kidney function. Linear regression models adjusted for age, sex, dyslipidemia, hypertension, log-UPCR, glomerular CKD etiology, CKD duration, calcium, phosphorus, and log-iPTH. Estimates are %increase in CIMT per 10% increase in metabolite level

Metabolite	Bile acid subpathway	Spearman correlation with eGFR*	Adjusted Model			Adjusted Model + log(eGFR)		
			Estimate (95%CI)	p	FDR	Estimate (95%CI)	p	FDR
taurocholate	Primary	-0.21 (p = 0.021)	0.69 (0.13, 1.24)	0.018	0.16	0.64 (0.08, 1.20)	0.028	0.25
3b-hydroxy-5-cholenoic acid	Secondary	0.29 (p = 0.001)	-1.55 (-2.54, -0.55)	0.004	0.047	-1.47 (-2.48, -0.44)	0.007	0.09
taurocholenate sulfate	Secondary	-0.24 (p = 0.009)	1.76 (0.69, 2.84)	0.002	0.047	1.67 (0.56, 2.79)	0.004	0.09
taurourso-deoxycholate	Secondary	-0.20 (p = 0.030)	0.71 (0.11, 1.31)	0.023	0.16	0.65 (0.04, 1.27)	0.040	0.27

There were 74 participants with 89 follow-up assessments. The associations of bile acid metabolites with CIMT were not replicated in the longitudinal analyses.

## Discussion

We showed that bile acid metabolites are associated with CIMT in children with CKD. Circulating bile acid levels are moderately correlated with eGFR. Three bile acid metabolites (taurocholate, taurocholenate sulfate, and tauroursodeoxycholate) had higher levels with eGFR decline and were associated with higher CIMT. Whereas, 3b-hydroxy-5-cholenoic acid had lower levels with eGFR decline and were associated with lower CIMT. These observations were significant in fully adjusted models at *p* < 0.05. This aligns with animal model and adult CKD data of bile acids effects on atherosclerosis in CKD.

Statistical caution is required when interpreting this data. Our sample is relatively small and the effect of each individual bile acid on CIMT is also small. Bile acids and eGFR are multicollinear. Although none of the associations were significant at FDR < 0.05 in the fully adjusted model, there is already extensive data describing the association of bile acids and atherosclerosis outside of children with CKD.

The association of bile acids with CIMT was not replicated with the longitudinal follow-up sample. The smaller number of person-visits likely reduced the power. New bias was also likely introduced, as CKiD participants who completed follow-up metabolomics assessments have previously been shown to represent a healthier phenotype, with higher eGFR at study entry, less proteinuria, fewer glomerular CKD etiologies, and lower prevalence of hypertension and dyslipidemia.

This is an important biologic relation to confirm in children with CKD. In the parallel disease model of cholestatic liver disease, research has progressed towards therapeutic modulation of circulating bile acids through intestinal, kidney proximal tubule, and hepatocyte uptake inhibitors [10]. More studies are needed to confirm if bile acids and their downstream metabolic signaling pathways are a viable therapeutic target for anti-atherosclerotic therapy in CKD [11].

## Data Availability

Investigators interested in accessing CKiD data may submit inquiries through the CKiD website listed in the acknowledgments.
